# Down‐regulation of TFAM increases the sensitivity of tumour cells to radiation via p53/TIGAR signalling pathway

**DOI:** 10.1111/jcmm.14350

**Published:** 2019-05-06

**Authors:** Xu Jiang, Jun Wang

**Affiliations:** ^1^ Key Laboratory of High Magnetic Field and Ion Beam Physical Biology Chinese Academy of Sciences Hefei China; ^2^ The University of Science and Technology of China Hefei China

**Keywords:** cell proliferation, mitochondrial superoxide, p53/TIGAR signalling, radio‐sensitivity, TFAM

## Abstract

Mitochondrial transcription factor A (TFAM) is a key regulator of mitochondria biogenesis. Previous studies confirmed that reduced TFAM expression sensitized tumours cells to chemical therapy reagents and ionizing irradiation (IR). However, the underlying mechanisms remain largely unknown. In this study, we identified that decreased expression of TFAM impaired the proliferation of tumour cells by inducing G1/S phase arrest and reducing the expression of E2F1, phospo‐Rb, PCNA and TK1. Furthermore, we proved that knockdown of TFAM enhanced the interaction between p53 and MDM2, resulting in decreased expression of p53 and the downstream target TIGAR, and thus leading to elevated level of mitochondrial superoxide and DNA double‐strand break (DSB) which were exacerbated when treated the cell with ionizing radiation. Those indicated that knockdown of TFAM could aggravate radiation induced DSB levels through affecting the production of mitochondria derived reactive oxygen species. Our current work proposed a new mechanism that TFAM through p53/TIGAR signalling to regulate the sensitivity of tumour cells to ionizing radiation. This indicated that TFAM might be a potential target for increasing the sensitization of cancer cells to radiotherapy.

## 1 INTRODUCTION

Mitochondria, the powerhouse of cell, regulate calcium homeostasis, glucose utilization, lipids biosynthesis and apoptosis.^1^, ^2^ The healthy mitochondria are responsible for maintaining cellular homeostasis. However, in tumour cells, due to mutations in oncogenes and tumour suppressor genes, mitochondrial roles have changed.^3^, ^4^, ^5^ The alterations in electron transport chain (ETC) result in oxidative stress through increasing cellular reactive oxygen species (ROS) levels, tumour cells invasion and proliferation.^6^ To sustain proliferation, tumour cells use the tricarboxylic acid (TCA) cycle to supply cancer cells with large amounts of macromolecular intermediates for biosynthesis.^7^ A dynamic feedback is the cancer cells have the ability to regulate signalling pathways to affect mitochondria, which in turn impact tumorigenesis.^8^, ^9^, ^10^

Human mitochondrial transcription factor A (TFAM), encoded by the nuclear gene *TFAM*, is required for mitochondrial DNA replication and transcription, which are essential for mitochondrial biogenesis.^11^, ^12^ The results from ONCOMINE database of cancer microarray assays show that TFAM is up‐regulated in a variety of tumour cell lines.^13^ Depletion of TFAM increases both Ca^2+^ and ROS levels, activates calcineurin‐mediated mitochondrial retrograde signalling, thus inducing expression of CFAP65 and PCK1 which participate in the change of cell morphology and cell proliferation.^14^ Down‐regulation of TFAM reduces mtDNA copy number, enhanced the sensitivity of tumour cells to chemotherapeutic drugs and increases the sensitivity of non‐small‐cell lung cancer cells to cisplatin by promoting ROS‐induced caspase‐dependent apoptosis.^15^, ^16^ The inhibition of TFAM in OSC‐2 cells results in reduced cell viability and strongly induces apoptosis after γ‐ray irradiation.^17^ Although previous studies provided evidence testify that TFAM is implicated in regulating tumour cells growth and their sensitivity to tumour therapy agents, but the underlying mechanisms remain to be uncovered, which may provide novel ways for cancer therapy.

P53 is involved in regulation of the cycle arrest, apoptosis and senescence.^18^ It not only interacts with the promotor of *TFAM* to activate *TFAM* transcription but also binds with TFAM to regulate cell death.^19^, ^20^, ^21^ However, whether TFAM can influence p53 has not been identified. As a transcription factor, p53 can regulate the expression of numerous target genes besides TFAM.^22^ TIGAR (TP53 Induced Glycolysis and Apoptosis Regulator), one of the p53‐inducible proteins, functions as a fructose‐2, 6‐bisphosphatase. It promotes the pentose phosphate pathway and helps to lower intracellular ROS.^23^, ^24^ ROS plays important roles in regulating cell signalling and homeostasis,^25^, ^26^ however, excessive amounts of ROS damages cellular components such as DNA, proteins and lipids, resulting in disturbance of cellular physiological status and cell death.^27^, ^28^ Ionizing radiation can effectively induce genetic mutagenesis and death of mammalian cells, making it a clinical way for cancer therapy. Elevated level of ROS is one of the mechanisms for radiation to inhibit the proliferation and promote death of tumour cells.^29^ Mitochondrial electron transport chain (ETC) is the key source of cellular ROS. Due to its direct regulation of ETC proteins, TFAM may affect the production of ROS and further influence cellular proliferation and death.

In this study, we aimed at investigating how TFAM affected the sensitivity of tumour cells to ionizing irradiation. We found that attenuated TFAM expression retarded tumour cells proliferation through inducing G1/S phase arrest. Decreased expression of TFAM resulted in inhibition of p53/TIGAR signalling, which further led to elevated mitochondrial superoxide production and DNA double‐strand breaks levels in irradiated tumour cells. These results brought new insight to understand the role of TFAM in regulating the radiation sensitivity of tumour cells, and were described in the following.

## 2 MATERIALS AND METHODS

### 2.1 Cell culture and radiation

The human tumour cell lines Hep G2, U‐2 OS and MCF7 were from ATCC (Manassas, VA, USA) and cultured in DMEM/F12 supplemented with 10% FBS at 37°C in a 5% CO_2_ incubator. Gamma ionizing irradiation (IR) was carried out in a Biobeam GM gamma irradiator (Leipzig, Germany) containing a caesium137 source with the dose rate of 3.27 Gy/min.

### 2.2 Chemicals and reagents

Puromycin and Nutlin‐3 were obtained from Selleck (Houston, TX, USA). Mito‐SOX Red were purchased from (Invitrogen, USA). The following primary antibodies were used: TFAM, β‐actin, PCNA, TIGAR, P53 (Santa Cruz, California, USA), p‐Rb (Ser807/811), Cleaved caspase‐7, γ‐H_2_A.X (Cell Signal Technology, MA, USA), TK1, E2F1 (Proteintech, Wuhan, China), PARP ( BD Biosciences, Franklin Lakes, NJ, USA). HRP‐conjugated secondary antibodies were purchased from Jackson ImmunoResearch Laboratories (Jackson ImmunoResearch Inc; West Grove, PA, USA). DNA primers were synthesized by General Biosystems (Chuzhou, China). *TFAM* shRNA and *TIGAR* siRNA were purchased from OriGene (Rockville, MD, USA).

### 2.3 Transfection of shRNA plasmids and siRNA

shRNA plasmid targeted to *TFAM* and scrambled shRNA plasmid were transfected into the cells by Roche X‐tremeGENE HP DNA Transfection Reagent according to the manufacturer's protocol. Medium containing 1μg/ml puromycin was used to select transfectants. Knockdown of TFAM was confirmed by determining the expression level of TFAM by western blotting and the mRNA level by Quantitative real‐time PCR. siRNA targeted to *TIGAR* and scrambled siRNA were transfected into cells by Lipofectamine 2000 transfection reagent according to the manufacturer's protocol. 36 hours post transfection, the expression of TIGAR was tested by western blotting.

### 2.4 Western blotting analysis

The cells were washed twice with ice‐cold PBS and then lysed with RIPA buffer containing protease inhibitors and protein phosphatase inhibitors. After incubated on ice for 30 minutes, the lysate was centrifuged at 13800 *g* for 10 minutes at 4°C, Protein concentration of the supernatant was determined using a BCA kit (Sangon Biotech, Shanghai, China). Protein samples were resolved by 10 or 12% SDS‐PAGE and transferred onto PVDF membrane (Roche Diagnostics GmbH; Mannheim, Germany). The membranes were blocked in TBST (0.1% Tween‐20) with 5% non‐fat milk at room temperature and hybridized with the appropriate primary antibodies dissolved in TBST containing 5% non‐fat milk overnight at 4°C. After washing three times with TBST, the membrane was hybridized with corresponding HRP‐conjugated secondary antibody for 2 hours at room temperature and washed another three times with TBST. The membrane was visualized by using the enhanced chemiluminescence substrate (BOSTER Biological Technology, Wuhan, China) in chemiluminescence image analyser Bands intensity was analysed by ImageJ software (NIH, Bethesda, MD, USA).

### 2.5 Quantitative real‐time PCR

Total RNA was extracted using RNAiso (Takara, Shiga, Japan). Quantitative RT‐PCR (qRT‐PCR) was undertaken using One Step SYBR® PrimeScript^TM^ PLUS RT‐PCR Kit (Takara) according to the *ΔΔCt* method. Reverse transcription was carried out at 42°C for 10 minutes. The primers used for qRT‐PCR analysis of mRNA levels were: *TFAM* forward: GCGCTCCCCCTTCAGTTTTG, reverse: GTTTTTGCATCTGGGTTCTGAGC; *β‐Actin* forward: CCTGGCACCCAGCACAAT, reverse: GGGCCGGACTCGTCATAC; the primer for *PCNA* mRNA analysis were forward: CAAGTAATGTCGATAAAGAGGAGG, reverse: GTGTCACCGTTGAAGAGAGTGG; *TK1* forward: AGCAGCTTCTGCACACATGACC reverse: CTCGCAGAACTCCACGATGTCA. Reaction parameters were: 95°C for 15 seconds, 52°C for 30 seconds and 72°C for 30 seconds, for 35 cycles. The mRNA level of *β‐Actin* was used as endogenous control.

### 2.6 Colony formation assay

A total of 300 cells were seeded in 60‐mm dish. After irradiation, the dishes were incubated for two weeks at 37°C in a 5% CO_2_ incubator for 20 days. Then the dishes were washed with PBS, fixed with a solution containing methanol: acetic acid (V/V = 9:1) for 30 minutes and subsequently stained with crystal violet for 30 minutes. The colonies containing more than 50 cells per colony was scored and plotted.

### 2.7 Cell proliferation and cell cycle analysis

For cell proliferation analysis, 5000 cells were seeded into each well of 24‐well cell culture plate. The number of the cells was counted every 24 hours for six days, then recorded and plotted. For cell cycle and apoptosis analysis, the cells were digested with EDTA free trypsin and washed twice with ice‐cold PBS. The cells were then fixed in 70% ice‐cold ethanol overnight at 4°C. On the next day, the cells were washed twice with ice‐cold PBS. After centrifugation, the pellets were resuspended with PBS containing 0.1% Triton X‐100, 25 μg/mL RNase A and 25 μg/mL propidium iodide, and incubated for 30 minutes at 37°C in the dark box. The cell cycle distribution and radiation induced apoptosis were analysed using FACStarPLUS.

### 2.8 Immunofluorescence staining

To detect the level of DNA double‐strand breaks, the cells were washed twice with PBS and fixed with 4% paraformaldehyde at room temperature for 15 minutes after radiation or siRNA transfection treatment. Then the cells were permeabilized with 0.5% Triton X‐100 at room temperature for 30 minutes, blocked with 1% BSA in PBST (0.1% Triton X‐100) at room temperature for 1 hour. The cells were then incubated with primary anti‐γ‐H_2_AX antibody diluted in PBST containing 1% BSA overnight at 4°C. The dishes were then washed three times with PBST for 15 minutes. AlexaFlour‐594 conjugated goat anti‐rabbit IgG secondary antibody was used to incubate samples at room temperature for 2 hours. After wash with PBST, cell nuclei were stained with DAPI. Images were captured under Olympus IX83 fluorescence microscope. Fluorescence intensity was analysed with ImageJ software.

### 2.9 Measurement of mitochondrial superoxide levels

To detect mitochondrial superoxide level, the cells were washed twice with warm PBS. Pre‐warmed solution buffer containing 5 μmol/L Mito‐SOX fluorescence probe was added into the dish and incubated at 37°C for 10 minutes according to the manufacturer's protocols. Then, the cells were washed with pre‐warmed PBS and images were captured under fluorescence microscope. The relative fluorescence intensity was analysed by ImageJ.

### 2.10 Immunoprecipitation assay

Total cell lysate was prepared using RIPA buffer containing protease inhibitors. After centrifugation, Protein G agarose beads slurry was added into the lysate and incubated at 4°C for 30 minutes on a rotator. After centrifugation at 0.1 *g* for 3 minutes at 4°C, the supernatant was transferred to a fresh tube. Primary antibody was added into the supernatant and incubated at 4°C for 12 hours with gentle agitation. Then, Protein G agarose beads slurry was added to capture the protein complex. After incubation at 4°C for 3 hours with gentle agitation, The sample was centrifuged at 0.06‐0.1 *g* for 30 seconds at 4°C. The supernatant was discard and the pellet was washed with RIPA buffer. Finally, SDS‐PAGE loading buffer was used to resuspend the immunoprecipitate for western blotting analysis.

### 2.11 Statistical analysis

Statistical data are expressed as the mean ± standard error from at least three independent experiments. Significant differences between two groups were determined by student's *t* test using the Graphpad Prism software. For multiple groups comparison, one‐way ANOVA analysis by spss software was used. *P* < 0.05 represented the difference was statistically significant.

## 3 RESULTS

### 3.1 TFAM knockdown inhibits cell proliferation and results in G1/S phase arrest

**Figure 1:**
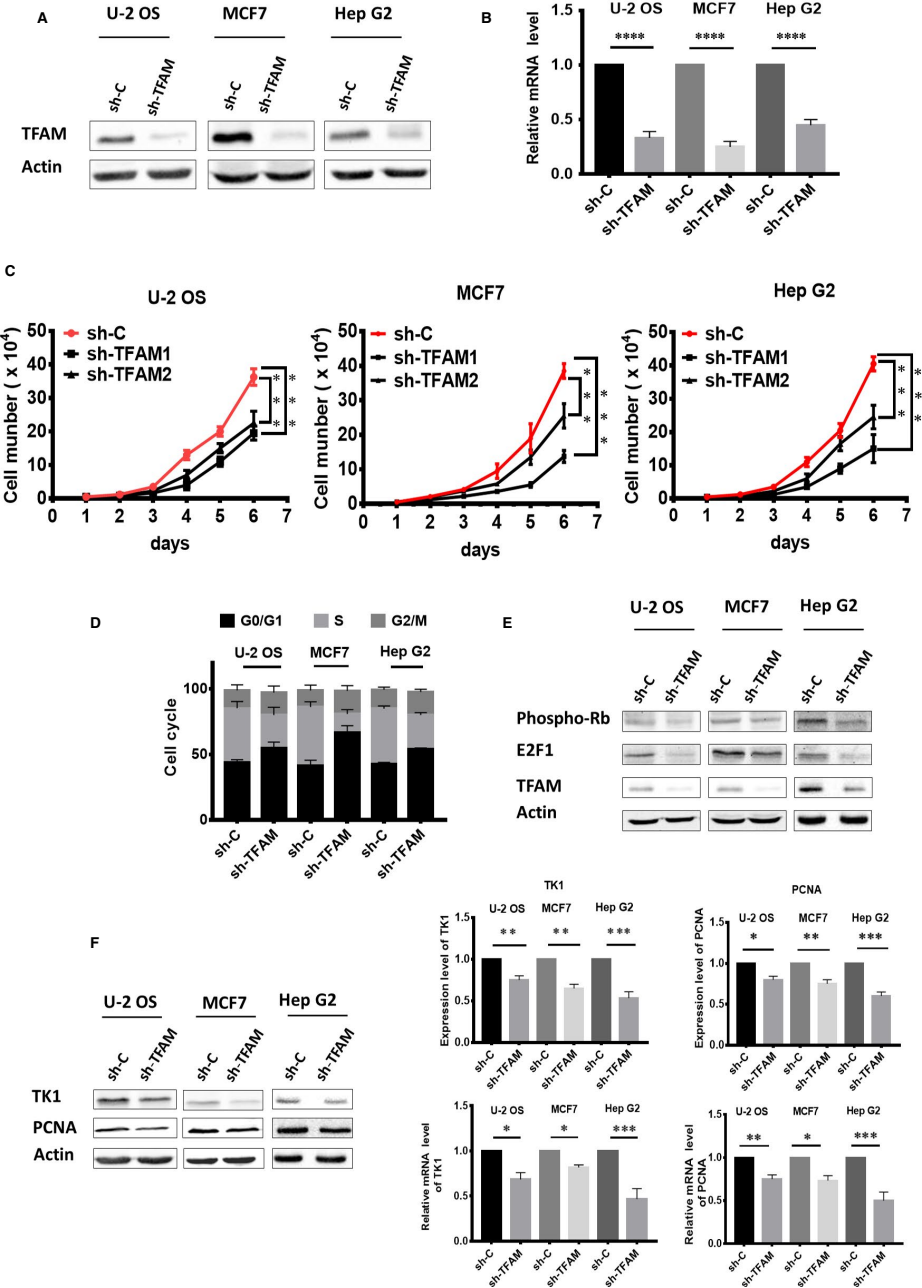
TFAM knockdown inhibits cell proliferation and results in G1/S phase arrest. A, Western blotting analysis of TFAM levels in three cancer cell lines after shRNA plasmid transfection; B, qPCR analysis of TFAM mRNA levels after shRNA plasmid transfection; C, Cell proliferation curves of the control and *TFAM* knockdown cells; D, Cell cycle distribution analysis by flow cytometry; E, Western blotting analysis of the proteins involved in the transition from G1 to S phase; F, Western blotting and quantitative reverse transcription PCR analysis of PCNA and TK1 in control and *TFAM* Knockdown cells. *, ** and *** represents *P* < 0.05, 0.01 and 0.001 respectively

To detect the function of TFAM on tumour cell proliferation, we established cell lines with low level expression of TFAM by transfecting shRNA plasmids in U‐2 OS, MCF7 and Hep G2 cells. Knockdown of TFAM was confirmed by detecting the protein level with western blotting and mRNA level with qRT‐PCR respectively (Figure 1A,B). Cell proliferation assay showed that down‐regulation of TFAM inhibited the proliferation of the three tumour cell lines (Figure 1C). Next, we detected whether the knockdown of TFAM affected cell cycle progression. Enhanced accumulation of G1 phase cells was observed for *TFAM* knockdown tumour cell lines based on flow cytometry results (Figure 1D), indicating TFAM knockdown led to G1/S phase arrest and attenuated cellular proliferation.

Related cell cycle regulators were further investigated. The levels of E2F1 and phosphorylated retinoblastoma‐associated protein (p‐Rb), which participate in controlling the transition from G1 to S phase in cells, were decreased in *TFAM* knockdown cells (Figure 1E). Moreover, both the protein and mRNA levels of PCNA and TK1, two downstream targets of E2F1, were down‐regulated in *TFAM* knockdown cell lines (Figure 1F), confirming that knockdown of TFAM resulted in G1/S phase arrest and attenuated cellular proliferation.

### 3.2 Knockdown of TFAM aggravates ionizing radiation induced DNA double‐strand breaks and cell death

**Figure 2:**
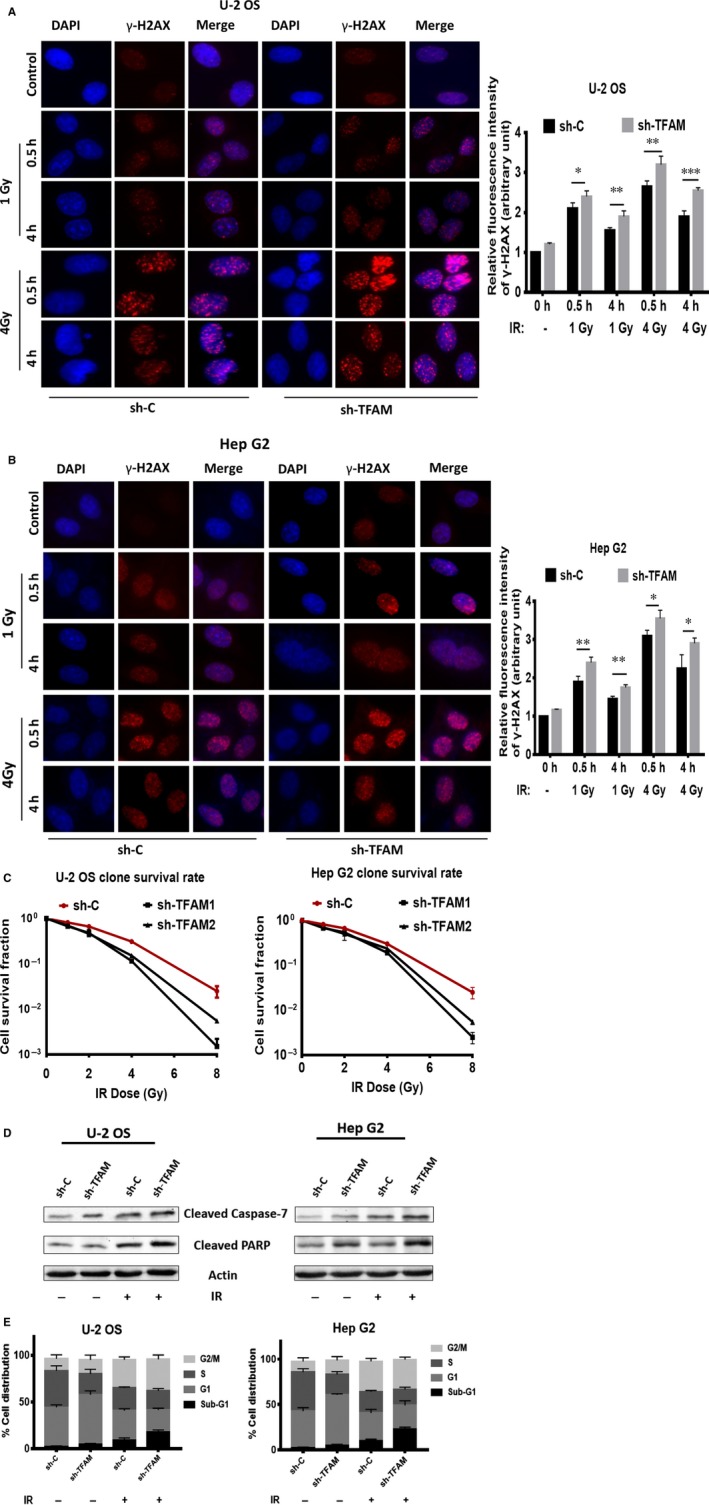
Knockdown of TFAM aggravates ionizing radiation induced DNA double‐strand breaks and cell death. A, The DNA double‐strand breaks levels of control and *TFAM* knockdown U‐2 OS cells after radiation; B, The DNA double‐strand breaks levels of control and *TFAM* knockdown Hep G2 cells after radiation; C, Clongenic assay of control and *TFAM* knockdown U‐2 OS and Hep G2 cells after radiation treatment. D, Western blotting analysis of cleaved caspase‐7 and PARP in irradiated or non‐irradiated control and *TFAM* knockdown U‐2 OS and Hep G2 cells; E, Flow cytometry analysis of apoptosis in irradiated or non‐irradiated control and *TFAM* knockdown U‐2 OS and Hep G2 cells. *, ** and *** represents *P* < 0.05, 0.01 and 0.001 respectively

Based on the above results that TFAM contributes to the proliferation of tumour cells, we next investigated it affects the cell death induced by ionizing radiation (IR), a well‐known method for clinical cancer therapy. The DNA double‐strand breaks (DSB), the most hazardous DNA damage, were detected in irradiated control and *TFAM* knockdown U‐2 OS and Hep G2 cells. As shown in Figure 2A,B, knockdown of TFAM resulted in slight increase of 20% of basal DSB levels in these two cell lines. 1 or 4 Gy gamma radiation was used to treat cells, and DSB levels were detected 0.5 or 4 hours post radiation. It could be observed that at all treatment conditions, knockdown of TFAM increase the DSB levels in U‐2 OS and Hep G2 cells. Furthermore, clonogenic assay was applied to evaluate the contribution of TFAM to radiation sensitivity in U‐2 OS and Hep G2 cells. As shown in Figure 2C, knockdown of TFAM led to decreased cell survival after ionizing radiation, which was in line with the DSB formation results. In addition, radiation induced apoptosis between the control and TFAM knockdown cells were compared. As shown in Figure 2D, 48 hours post 4 Gy radiation, the levels of cleaved caspase‐7 and PARP in TFAM knockdown U‐2 OS and Hep G2 cells were higher than those observed in the irradiated control cells. Apoptosis rate was evaluated by calculating the fraction of cells at sub‐G1 phase. As shown in Figure 2E, for control U‐2 OS cells, the apoptosis rate in non‐irradiated cell was around 2%, and was increased to 10% after 4 Gy irradiation. For TFAM knockdown U‐2 OS cells, the apoptosis rates were around 5% and 20% respectively. For control Hep G2 cells, the apoptosis rate in non‐irradiated cell was around 2%, and was increased to 12% after 4 Gy irradiation. For TFAM knockdown Hep G2 cells, the apoptosis rates were around 6% and 25% respectively. These results indicated that knockdown of TFAM‐sensitized cells to radiation.

### 3.3 Elevated mitochondrial superoxide level in TFAM knockdown caused DNA damage

**Figure 3:**
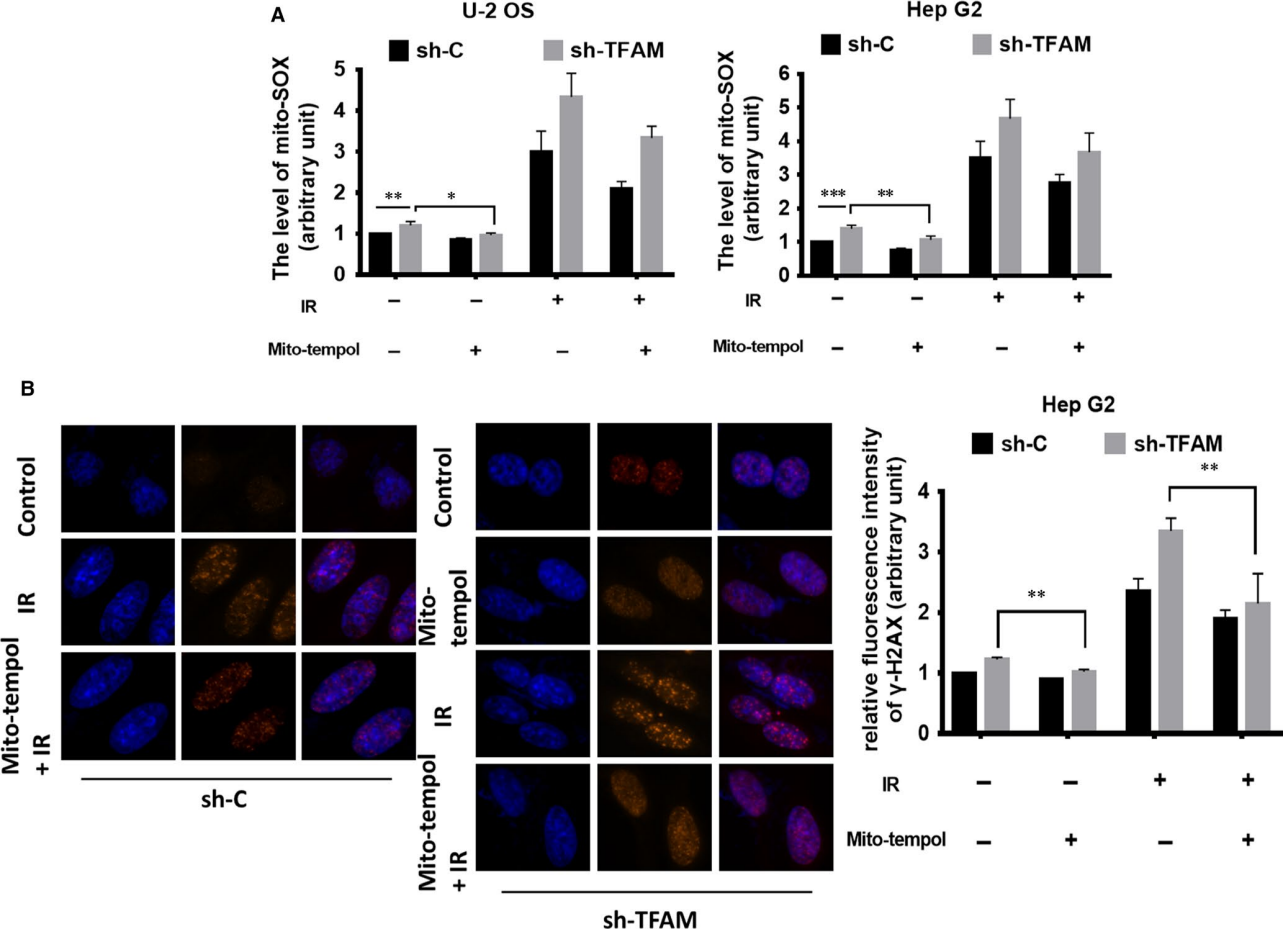
Elevated mitochondrial superoxide level in TFAM knockdown caused DNA damage. A, The levels of mitochondrial superoxide in control and *TFAM* knockdown U‐2 OS and Hep G2 cells exposed to radiation with or without mito‐tempol pre‐treatment; B, The DNA double‐strand breaks levels of control and *TFAM* knockdown Hep G2 cells after radiation with or without mito‐tempol pre‐treatment. *, ** and *** represents *P* < 0.05, 0.01 and 0.001 respectively

TFAM controls the biogenesis of mitochondria. We therefore detected cellular mitochondrial superoxide levels in the control and TFAM knockdown cells. As shown in Figure 3A, down‐regulation of TFAM resulted in slight, around 15%, elevation of mitochondrial superoxide. After 4 Gy γ radiation, the levels of mitochondrial superoxide in both the control and *TFAM* knockdown U‐2 OS and Hep G2 cells increased significantly (Figure 3A). Besides, knockdown of TFAM exacerbated mitochondrial superoxide production in irradiated cells, for U‐2 OS, increased by around 30%, and for Hep G2, increased by around 25%. Mito‐tempol is a specific mitochondrial superoxide scavenger. It partially inhibited the production of mitochondrial superoxide in irradiated cells (Figure 3A). By using mito‐tempol, we next investigated the relationship between mitochondrial superoxide levels and radiation induced DSB levels in control and *TFAM* knockdown cells. As shown in Figure 3B, pre‐treatment with mito‐tempol attenuated 4 Gy γ radiation induced DSB levels at 4 hours after radiation in Hep G2 cells. These results indicated that radiation induced mitochondrial superoxide was a DNA damaging agent, and TFAM knockdown enhanced mitochondrial superoxide and DSB levels.

### 3.4 TIGAR is involved in enhanced DSB formation in *TFAM* knckdown cells

**Figure 4:**
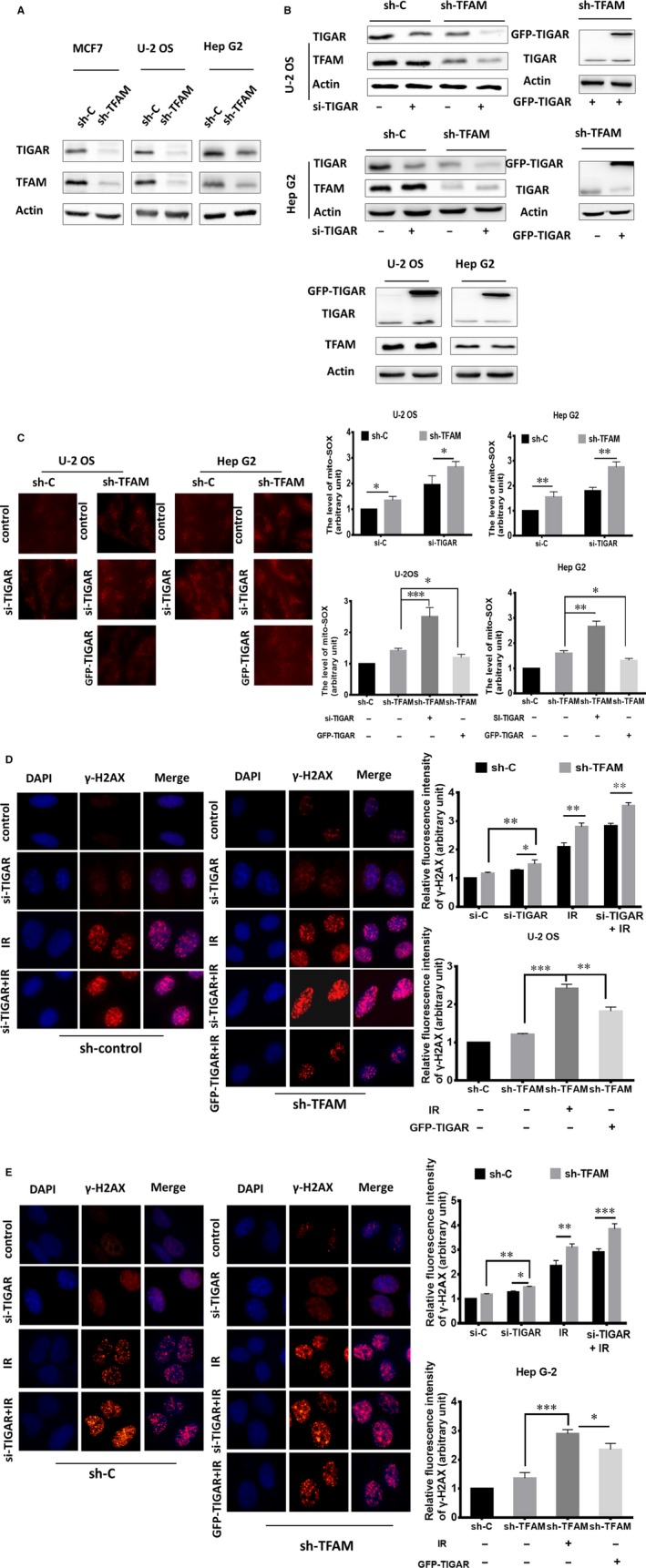
TIGAR is involved in enhanced DSB formation in *TFAM* knockdown cells. A, Western blotting analysis of TIGAR expression in *TFAM* knockdown MCF‐7, U‐2 OS and Hep G2 cells; B, Western blotting analysis of TIGAR levels in control and *TFAM* knockdown U‐2 OS and Hep G2 cells after transfection with *TIGAR* si‐RNA or TIGAR overexpression plasmid; C, Fluorescence microscopic analysis of mitochondrial superoxide levels in control and *TFAM* knockdown U‐2 OS and Hep G2 cells; (D, E) The DNA double‐strand breaks levels in irradiated control and *TFAM* knockdown U‐2 OS and Hep G2 cells after transfection with *TIGAR* si‐RNA or TIGAR overexpression plasmid respectively. *, ** and *** represents *P* < 0.05, 0.01 and 0.001 respectively

TIGAR was reported to be an anti‐oxidative protein. Since TFAM knockdown resulted in elevated mictochondrial superoxide, we then checked whether the enhanced production of mictochondrial superoxide and DSB in *TFAM* knockdown cells were due to deregulation of TIGAR. Western blotting analysis showed that the levels of TIGAR in *TFAM* knockdown MCF7, U‐2 OS and Hep G2 cells were significantly lower compared to the corresponding control cells (Figure 4A). Since TIGAR was reported to be an anti‐oxidative protein, we then checked whether the enhanced production of mictochondrial superoxide and DSB in *TFAM* knockdown cells were due to deregulation of TIGAR.

Furthermore, knockdown of TIGAR expression by siRNA in control or *TFAM* knockdown U‐2 OS and Hep G2 cells enhanced mitochondrial superoxide levels (Figure 4B,C), augmented by around 30%. We then overexpressed TIGAR in *TFAM* knockdown U‐2 OS and Hep G2 cells. As shown in Figure 4B,C, overexpression of TIGAR did not obviously affect the expression TFAM, but largely reduced the levels of mitochondrial superoxide, about half of the that were detected in *TFAM* knockdown cells. These results indicated that TFAM knockdown decreased TIGAR expression, which exacerbated mitochondrial superoxide production.

Next, we tested the effects of TIGAR on ionzing radiation (IR) induced DSB levels. siRNA targeted to TIGAR and scrambled siRNA were transfected into *TFAM* stable knockdown cells and control cells. Then, the cells were irradiated by 4 Gy γ ray. Half an hour later, DSB levels were detected. The inhibition of *TIGAR* caused DNA damage and aggravated it in U‐2 OS and Hep G2 KD cells (Figure 4D,E). When we transfected TFAM knockdown cells with GFP‐TIGAR overexpression plasmid to activate TIGAR (Figure 4B), it was effective to reduce the formation of γ‐H2AX foci after γ‐radiation (Figure 4D, 4). As the statistical data shown, knockdown of *TIGAR* caused around 25% increase of basal DSB levels in both the control and *TFAM* knockdown U‐2 OS and Hep G2 cells. Upon radiation, *TIGAR* knockdown aggravated DSB levels. On the contrary, in TIGAR overexpressed *TFAM* knockdown U‐2 OS and Hep G2 cells, radiation induced DSB levels were attenuated, 20% decrease was observed. Together with the mitochondrial superoxide results, TFAM knockdown exacerbated radiation induced DSB levels through down‐regulating TIGAR expression and augmenting mitochondrial superoxide levels.

### 3.5 TFAM knockdown down‐regulates p53/TIGAR signal pathway

**Figure 5:**
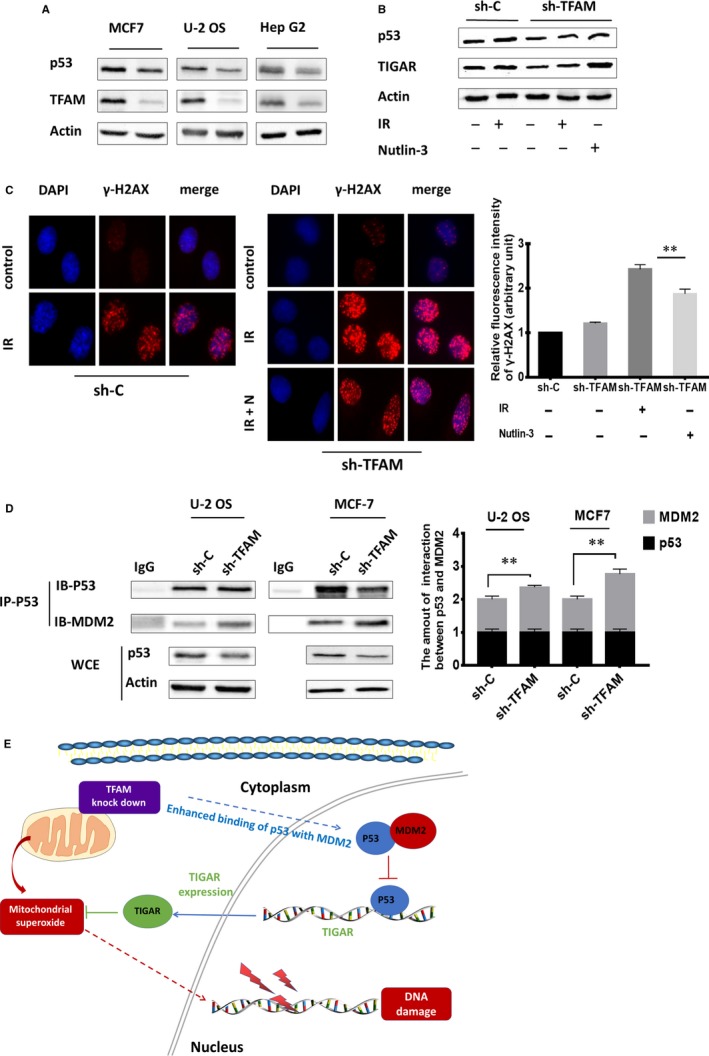
TFAM knockdown down‐regulates p53/TIGAR signal pathway. A, Western blotting analysis of p53 levels in control and *TFAM* knockdown MCF7, U‐2 OS and Hep G2 cells; B, The protein levels of p53 and TIGAR in control and *TFAM* knockdown U‐2 OS cells after radiation and nutlin‐3 treatment; C, The DNA double‐strand breaks levels in control and *TFAM* knockdown U‐2 OS cells after radiation and nutlin‐3 treatment; D, Analysis of the binding between p53 and MDM2 in control and *TFAM* knockdown U‐2 OS and MCF7 cells by immunoprecipitation and immunoblotting analysis. *, ** and *** represents *P* < 0.05, 0.01 and 0.001 respectively; E, Schematic illustration of the proposed model. TFAM knockdown enhanced the interaction between p53 and MDM2, resulting in the down‐regulation of p53 and lowered expression of TIGAR. This further increased the accumulation of mitochondrial superoxide ROS and induced the DNA damage. And therefore, after exposure to irradiation, DNA damages in irradiated cells will be exaggerated

TIGAR is a downstream target of p53. We therefore examined the level of p53 in *TFAM* knockdown cells. As expected, the expression levels of p53 in *TFAM* knockdown MCF7, U‐2 OS and Hep G2 cells were lower than those in the corresponding control cells (Figure 5A). We further treated control and *TFAM* knockdown U‐2 OS with 4 Gy γ radiation and 10μmol/L p53 activator nutlin‐3 (N), and then detected the expression levels of p53 and TIGAR. As shown in Figure 5B, radiation increased the expression of p53 and TIGAR in U‐2 OS cells. Nutlin‐3 further enhanced p53 and TIGAR expression, confirming the p53/TIGAR signalling axis in *TFAM* knockdown cells. Since we have identified that decreased expression of both TFAM and TIGAR exacerbated radiation induced DSB levels, we then checked whether increasing the p53 levels in *TFAM* knockdown cells attenuated DSB levels in irradiated cells. As shown in Figure 5C, in 4 Gy γ irradiated *TFAM* knockdown U‐2 OS cells, nutlin‐3 treatment decreased DSB levels by around 25%, showing the protective role of p53/TIGAR axis in the context of *TFAM* deficiency.

We then investigated the reason for reduced p53 levels in* TFAM* knockdown cells. Human mouse double minute 2 homolog (MDM2) functions as an E3 ubiquitin‐protein ligase that mediates ubiquitination of p53, leading to its degradation by the proteasome. The interaction between p53 and MDM2 was evaluated, respectively, in control and *TFAM* knockdown U‐2 OS and MCF‐7 cells by immunoprecipitation and immunoblotting. As shown in Figure 5D, by analysing the whole‐cell extract (WCE), knockdown of TFAM enhanced the interaction between p53 and MDM2. This indicated that *TFAM* knockdown down‐regulated p53/TIGAR signal pathway, which resulted in enhanced mitochondrial superoxide accumulation and DSB levels in irradiated cells (Figure 5E).

## 4 DISCUSSION

Our results indicated that tumour cells lacking TFAM exhibited proliferation retardation and G1/S phase cell cycle arrest. TFAM knockdown results in reduced expression of E2F1, a typical activator of the E2F family functions in the control of cell cycle progression from G1 to S phase. E2F1 holds inactive when associated with Rb, a transcription repressor of E2F1.^30^ In its activatory state, E2F1 can bind the promoters of target genes and induce transcription that result in various outcomes including cell cycle progression and DNA repair.^31^, ^32^ We demonstrated that down‐regulation of E2F1 and p‐Rb associated with TFAM knockdown further resulted in the reduction in PCNA and TK1, and blocked the cell cycle, correlated the function of TFAM with abnormal cell proliferation. In non‐small‐cell lung cancer ( NSCLC) cell lines, the suppression of TFAM inhibited cell proliferation through activating ROS induced c‐Jun amino‐terminal kinase (JNK) and p38 MAPK signalling pathway.^16^ And overexpression of miR‐200a to decrease TFAM protein expression resulted in attenuated cell proliferation,^33^ which is consistent with our result. However, depletion of TFAM in epidermal progenitor showed a profound reduction in mitochondrial DNA and respiratory chain complexes, only slight effect on the proliferation and differentiation was observed.^34^ This may due to the differences of cellular background. Ueta *et al* reported that TFAM knockdown reduces DNA repairing associated molecules and increases the sensitivity of cells to radiation by affecting cell apoptosis.^17^ Besides, it was reported that suppression of TFAM notably sensitized tumour cells to cisplatin and doxorubicin. Together with our result, TFAM is a candidate target for increase the efficiency of cancer chemo‐ or radiotherapy.

Mitochondrial superoxide is one of the cellular reactive oxygens. It is generated endogenously in the process of mitochondrial oxidative phosphorylation, and is known for playing both deleterious and beneficial functions in normal cellular physiological status.^35^, ^36^ TFAM knockdown causes mitochondrial membrane potential (MMP) depolarization and stimulates the production of mitochondrial superoxide.^16^, ^37^ TIGAR acts as a negative regulator of glycolysis and enables cells to scavenge ROS.^23^, ^38^ TIGAR‐knockdown results in the loss of colony formation capacity and delayed DNA repair of glioblastoma cells, leading cells to undergo morphological changes.^39^ However, overexpression of TIGAR in glioblastoma reduces cell death induced by glucose and oxygen restriction and enhances cellular defensed against ROS.^40^ It has also been reported that under hypoxia, a fraction of TIGAR relocalizes to mitochondria and executes its function by limiting mitochondrial ROS levels and protecting cells from death.^41^ In this work, TFAM knockdown repressed the expression level of TIGAR, leading to increased level of mitochondrial superoxide and DNA double‐strand breaks (DSB) induced by radiation. Transfection with *TIGAR* siRNA further intensified mitochondrial superoxide level and exacerbated DNA double‐strand breaks in TFAM knockdown cells. However, this was reversed by mitochondrial superoxide specific scavenger mito‐tempol, indicating the elevation of mitochondrial superoxide level due to TIGAR down‐regulation in irradiated TFAM knockdown cells was partially responsible for the increased DSB levels. And this was further supported by the result that overexpression of TIGAR in TFAM knockdown cells attenuated the levels of DSB and mitochondrial superoxide after radiation. Besides, it has been reported that TFAM can protect mtDNA from impairment by ROS, and higher level of TFAM can resist the radiation.^17^, ^42^ Together with our current results, it can be inferred that TFAM is essential for cellular redox homeostasis and cellular proliferation, and TIGAR is one of the mediator.

P53, as a powerful tumour suppressor gene, is involved in various cellular processes, including differentiation, apoptosis, senescence, metabolism and DNA repair.^43^ p53 can combine TFAM to form p53/TFAM/mtDNA complexes and interact with mitochondrial DNA polymerase to promote the replication and base excision repair of mtDNA.^44^, ^45^ Besides, loss of p53 leads to mitochondrial DNA depletion and altered mitochondrial reactive oxygen species homeostasis.^46^ Previous studies proved that p53 can affect the mitochondrial homeostasis, regulate the level of TFAM.^47^ However, the impact of TFAM on p53 is still not clear. We demonstrated here that TFAM knockdown notably down‐regulated the expression of p53. TIGAR is a downstream target of p53, and its expression under stress conditions was co‐related with the level of p53.^23^, ^48^ It was observed in our work that both p53 and TIGAR were down‐regulated in TFAM knockdown cells. And addition of p53 activator nutlin‐3 restored p53 and TIGAR levels, and alleviated the DSB levels in TFAM knockdown cells irradiated or not, confirming the direct regulation of TIGAR by p53. These results indicated the signalling way that inhibited TFAM resulted in lowering expression of p53/TIGAR axis, and therefore the enhancement of mitochondrial superoxide levels in irradiated cancer cell lines, which augmented the cell killing efficiency of radiation. P53 is negatively regulated by MDM2 via direct binding, and further ubiquitination and degradation.^49^, ^50^ We observed in TFAM knockdown cells, the binding of p53 with MDM2 was enhanced, which was an explanation for the down‐regulation of p53.

In conclusion, our current work revealed a mechanism about the impact of mitochondrial function regulator TFAM on the sensitivity of tumour cells to ionizing radiation. Lowering the expression of TFAM in cancer cell lines resulted in cell cycle arrest at G1/S phase, attenuated cellular proliferation, enhanced DNA damage and cell killing levels by radiation. These were partially due to the down‐regulation of p53/TIGAR signalling axis which functioned to scavenge mitochondrial superoxide level and maintain cellular redox homeostasis. Our results provided information about how mitochondria affected cellular oxidative stress and suggested that TFAM might be a sensitizing target in cancer radiotherapy.

## CONFLICT OF INTEREST

All authors have disclosed that they do not have any conflict of interest.

## DATA AVAILABILITY STATEMENT

The data used to support the findings of this study are included within the article.
